# Risk and characteristics of tuberculosis after anti-tumor necrosis factor therapy for inflammatory bowel disease: a hospital-based cohort study from Korea

**DOI:** 10.1186/s12876-021-01973-5

**Published:** 2021-10-20

**Authors:** Jae Yong Lee, Kyunghwan Oh, Hee Seung Hong, Kyuwon Kim, Seung Wook Hong, Jin Hwa Park, Sung Wook Hwang, Dong-Hoon Yang, Byong Duk Ye, Jeong-Sik Byeon, Seung-Jae Myung, Suk-Kyun Yang, Ho-Su Lee, Kyung-Wook Jo, Sang Hyoung Park

**Affiliations:** 1 Department of Gastroenterology, Asan Medical Center, University of Ulsan College of Medicine, 88 Olympic-ro 43-gil, Songpa-gu, Seoul, 05505 Korea; 2 Department of Biochemistry, University of Ulsan College of Medicine, Asan Medical Center, 88 Olympic-ro 43-gil, Songpa-gu, Seoul, 05505 Korea; 3 Department of Pulmonary and Critical Care Medicine, Asan Medical Center, University of Ulsan College of Medicine, 88 Olympic-ro 43-gil, Songpa-gu, Seoul, 05505 Korea

**Keywords:** Inflammatory bowel diseases, Tuberculosis, Tumor necrosis factor inhibitor, Latent tuberculosis, Risk factors

## Abstract

**Background:**

Anti-tumor necrosis factor (TNF) treatment for inflammatory bowel disease (IBD) increases the risk of tuberculosis (TB) infection. In the present study, we analyzed the clinical characteristics and risks of TB in Korean patients with IBD who received anti-TNF treatment.

**Methods:**

The study included patients with IBD who were treated using anti-TNF agents between January 2001 and June 2018 at the Asan Medical Center. Overall, 1434 patients with ulcerative colitis or Crohn’s disease were enrolled. We calculated the incidence of active TB infection after anti-TNF treatment and compared the clinical characteristics of the TB group with those of the non-TB group.

**Results:**

Twenty-one patients (1.46%) developed active TB infection, and the incidence rate of active TB was 366.73 per 100,000 person-years. In total, 198 patients (14.9%) were positive for latent tuberculosis infection (LTBI), of whom only eight (4%) did not complete LTBI treatment. The age at which the anti-TNF therapy was started was significantly higher in the TB group than in the non-TB group (HR 1.041, 95% CI 1.014–1.069, *p* = 0.002), and as age increased, so did the incidence rate of active TB infection (linearity *p* < 0.001). There was no significant difference in the incidence rate of LTBI between the TB and non-TB groups (HR 0.896, 95% CI 0.262–3.066, *p* = 0.862).

**Conclusions:**

In patients with IBD, the incidence rate of TB increased with age at anti-TNF therapy initiation. Active treatment of LTBI may lower the incidence of TB in patients with IBD who are to undergo anti-TNF therapy.

## Background

Inflammatory bowel disease (IBD) is a disorder involving abnormal chronic inflammation in the gastrointestinal tract, which is caused by immune dysregulation. The disorder encompasses ulcerative colitis (UC) and Crohn's disease (CD) [1, 2]. The number of patients with IBD has increased recently in Korea and other Asian countries, where the disorder was once considered rare [3–7]. Immune dysregulation in IBD results in overproduction of tumor necrosis factor (TNF)-α, and monoclonal antibodies targeting TNF-α can suppress the abnormal immune response in IBD [8]. Such anti-TNF therapy has proven effective in both induction and maintenance therapy in patients with IBD, so the use of the anti-TNF therapy is increasing worldwide [9–11].

However, anti-TNF therapy increases the risk of infection [12, 13]. Tuberculosis (TB) is one infection associated with anti-TNF therapy, which is thought to inhibit the formation of granuloma and thus prevent the suppression of TB activation [14]. Because anti-TNF therapy increases the risk of active TB development through the reactivation of latent TB [15, 16], guidelines recommend diagnosing and treating latent tuberculosis infection (LTBI) before starting anti-TNF therapy [12, 16, 17]. Although the incidence of TB is decreasing worldwide, interest in the disease is increasing in Korea, which still has a higher incidence of TB and LTBI than more developed countries [18–20].

Several studies have investigated the development of TB after anti-TNF therapy in patients with IBD [14, 18, 21, 22]. However, most such research was conducted in multi-institution cohort studies, in which each institution often applied different standards of diagnosis, treatment, and follow-up. Therefore, it is worthwhile to analyze the risks and characteristics of TB infection in patients who have undergone standardized management of IBD and LTBI before anti-TNF therapy in Korea. In the present study, we used a well-established hospital-based cohort in Korea to evaluate the incidence, characteristics, and risk factors of TB in patients with IBD who received anti-TNF therapy.

## Methods

### Study design

**Fig. 1:**
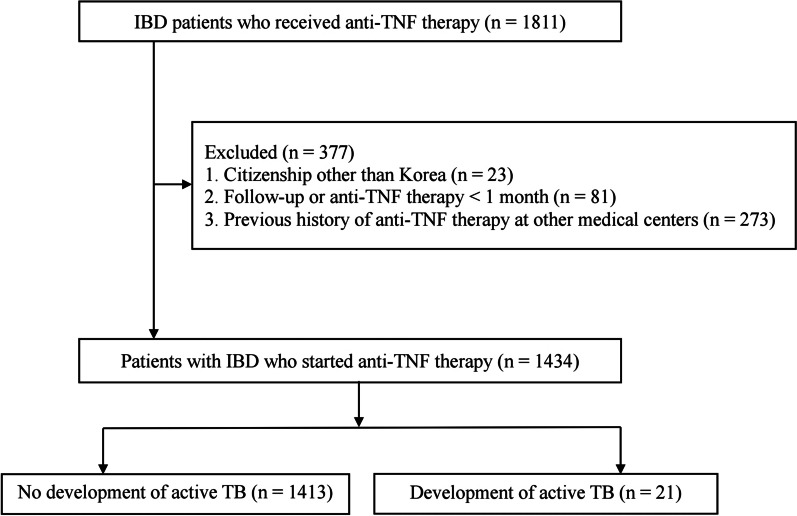
Study flow diagram. IBD, inflammatory bowel disease; TNF, tumor necrosis factor; TB, tuberculosis

The present retrospective study was conducted at the Asan Medical Center, a tertiary medical center in Korea. Between January 2001 and June 2018, 1811 patients with IBD (1386 with CD and 425 with UC) were prescribed anti-TNF agents in our institution. During the study period, the anti-TNF agents used to treat IBD included infliximab, adalimumab, and golimumab. According to the following criteria, 377 patients were excluded: (1) citizenship of a state other than Korea, (2) duration of follow-up or anti-TNF therapy < 1 month, and (3) previous history of anti-TNF therapy at other medical centers. Consequently, 1434 patients with IBD were eligible for the analysis (Fig. 1).

The following patient information was collected from the medical records: age, sex, type of IBD, age at IBD diagnosis, smoking status, results of LTBI screening, history of TB, anti-TNF agent type, age at the start of anti-TNF therapy, and concomitant medication, such as steroids and immunomodulators. The study protocol was approved by the Institutional Review Board of Asan Medical Center (IRB No. 2018-1178).

### Screening and management of LTBI

According to Korean guidelines, screening for LTBI is recommended before the initiation of anti-TNF therapy [23]. Most patients subject to LTBI screening were referred to TB specialists for work-up and management. They were checked for past medical history of TB infection and any symptoms suggestive of TB. LTBI tests consisted of simple chest X-rays, the tuberculin skin test (TST), and/or the interferon gamma release assay (IGRA). The TST was carried out following the Mendel–Mantoux method using purified protein derivative (PPD). An induration with a diameter of ≥ 10 mm was considered positive 48–72 h after PPD inoculation on the forearm [24]. The IGRA was performed using QuantiFERON®-TB Gold In-Tube (QFT-GIT; Cellestis, Carnegie, VIC, Australia) and/or T-SPOT®.TB (Oxford Immunotec, Abingdon, UK). The test results were defined according to the manufacturer's instructions. The subjects were considered to have LTBI based on the following criteria: (1) patients with a positive TST or IGRA test in the absence of clinical or radiological signs of active TB or (2) those with no history of TB treatment and lesions suggesting spontaneously healed TB on chest X-ray [23, 24].

Patients with a positive LTBI underwent TB prophylaxis, which usually started with isoniazid and rifampin for 3 months. If the patients had any comorbid disease that contraindicated this combination therapy, or if adverse effects occurred, rifampin for 4 months or isoniazid for 9 months was administered. Patients who completed LTBI screening were followed up during their anti-TNF therapy, regardless of whether they were positive for LTBI. At regular intervals, chest radiography was carried out, and the patients were checked for symptoms or signs suggesting TB.

### Statistical analysis

Hazard ratios (HRs) and 95% confidence intervals (CIs) were calculated using Cox proportional hazards models to evaluate the risk factors for TB development. The incidence rate (IR) of TB among patients with IBD was calculated as the number of patients with TB per 100,000 person-years of the population. The 95% CI of the IR was based on the Poisson probabilities of the corresponding patients, and the linearity *p* value of IR was calculated based on the log-binomial model. The TB-free survival probability was constructed using the Kaplan–Meier method. All *p* values < 0.05 were considered statistically significant. Statistical analyses were performed using the R program, version 4.0.3 (The R Foundation for Statistical Computing, Vienna, Austria).

## Results

### Baseline characteristics of the study patients

A total of 1434 patients with IBD who started anti-TNF therapy were included. The baseline characteristics of the patients who underwent anti-TNF therapy are shown in Table 1. The mean age at anti-TNF therapy initiation was 31.35 ± 13.59 years, and the mean follow-up period was 48.54 months. Among the patients, 940 (65.6%) were male and 1102 (76.8%) had CD. There were 991 (69.1%) non-smokers. Three types of anti-TNF agents were used: infliximab, adalimumab, and golimumab. There were 1259 patients (87.8%) who were given only one agent, and most of them used infliximab. There were 166 patients (11.6%) who used two drugs and three (0.2%) who used three drugs.

**Table 1:** Baseline characteristics of 1434 patients who underwent anti-TNF therapy

Characteristics	
Age of IBD diagnosis (years)	25.81 ± 12.45
Age at anti-TNF therapy initiation (years)	31.35 ± 13.59
Follow up (months)	48.54 ± 36.48
Sex (male:female)	940:494
IBD type (UC:CD)	332 (23.2%):1102 (76.8%)
History of smoking (no:yes)	991 (69.1%):443 (30.9%)
Anti-TNF agents	
One	1259 (87.8%)
Infliximab	939 (65.5%)
Adalimumab	311 (21.7%)
Golimumab	9 (0.6%)
Two	172 (12.0%)
Infliximab, Adalimumab	166 (11.6%)
Infliximab, Golimumab	2 (0.1%)
Adalimumab, Golimumab	4 (0.3%)
Three	3 (0.2%)
LTBI diagnosis	
Done	1333 (93.0%)
Negative	1135 (85.1%)
Positive	198 (14.9%)
Completion of TB prophylaxis	190 (96%)
Failure to complete TB prophylaxis	8 (4.0%)
Not done, other	101 (7.0%)

TNF, tumor necrosis factor; IBD, inflammatory bowel disease; UC, ulcerative colitis; CD, Crohn’s disease; LTBI, latent tuberculosis infection; TB, tuberculosis

With regard to the tests for LTBI, 939 patients underwent the TST and 1328 were tested using the IGRA. There were 966 patients who underwent both the IGRA and the TST. In total, 1333 patients (93.0%) completed LTBI screening, of whom 198 (14.9%) were positive for LTBI. Among them, 166 patients (83.8%) underwent TB prophylaxis according to our protocol. Twenty-four patients (12.1%) had a history of TB medication for active TB or to identify intestinal TB and did not require additional TB prophylaxis. Only 8 patients (4.0%) did not complete TB prophylaxis.

### Clinical characteristics of patients who developed active TB during anti-TNF therapy

Twenty-one patients with IBD were diagnosed with TB during anti-TNF therapy. The proportion of active TB development was 1.46% and the incidence rate was 366.73 per 100,000 person-years. The baseline characteristics of the patients who developed TB are described in Table 2. There were six patients with UC and 15 with CD, and there were 12 male and nine female patients. The median duration from the start of anti-TNF therapy to the diagnosis of active TB infection was 14 months (range 1–95). One patient had been diagnosed and treated for TB in the past. Seventeen patients developed TB while using infliximab. Four patients diagnosed with TB had changed to adalimumab or had received adalimumab from the beginning. Three of the patients with TB had positive LTBI screening, and had completed TB prophylaxis. Fourteen of the patients with TB received other immunosuppressants including steroids, azathioprine, and methotrexate within 3 months prior to TB diagnosis. Diabetes, chronic renal failure, and acquired immunodeficiency syndrome, which can influence the morbidity of TB, were not identified in any of the patients with TB. There were 18 cases (85.7%) of TB involving the lung and eight (38.1%) of TB involving other organs. In 20 patients, the TB was completely cured, while one patient was undergoing treatment at the end of the study.

**Table 2:** Characteristics of patients who developed active tuberculosis during anti-TNF therapy

No	IBD	Sex	Age of anti-TNF (years)	Age of TB (years)	Interval to TB diagnosis (months)	Anti-TNF agents	TST	IGRA	Previous TB treatment	LTBI	TB prophylaxis	Smoking	TB site
1	UC	M	67	68	2	IFX	ND	(−)	No	(−)	ND	Ex	Lung
2	UC	M	66	67	10	IFX	(−)	(−)	No	(−)	ND	Ex	Lung + peritoneum
3	UC	F	42	47	55	IFX	(−)	(−)	No	(−)	ND	(−)	Lung
4	UC	M	53	53	2	IFX	ND	(−)	No	(−)	ND	Ex	Lung
5	UC	M	46	47	14	IFX > ADA	ND	(+)	No	(+)	INH + RFP	Ex	Lung + pleura
6	UC	M	60	60	4	IFX	(−)	(−)	No	(−)	ND	(+)	pleura + pericardium
7	CD	F	33	33	3	IFX	(−)	(−)	No	(−)	ND	Ex	Lung
8	CD	M	59	59	3	IFX	(−)	(−)	No	(−)	ND	Ex	Lung
9	CD	M	26	29	34	ADA	(−)	(+)	No	(+)	INH + RFP	(−)	Lung
10	CD	F	26	30	50	IFX > ADA	(−)	(−)	No	(−)	ND	(−)	Lung
11	CD	M	23	25	27	IFX	ND	(−)	No	(−)	ND	(−)	Lung + LN
12	CD	F	32	32	4	IFX	ND	ND	No	Unknown	ND	(−)	Spleen + LN
13	CD	F	22	25	42	IFX	(−)	(+)	No	(+)	RFP	(−)	Lung
14^a^	CD	F	37	42	67	IFX	(−)	(−)	No	(−)	ND	(+)	Lung + pleura
15	CD	M	24	32	95	IFX	(−)	(−)	ITB^b^	(−)	ND	(+)	Lung + pleura
16	CD	M	29	30	5	IFX	(−)	Indeterminate	No	(−)	ND	(−)	Lung
17	CD	F	44	45	14	IFX	(−)	(−)	No	(−)	ND	(−)	Lung
18	CD	M	41	43	17	IFX	(−)	(−)	Lung	(−)	ND	(−)	Lung
19	CD	F	41	43	24	IFX > ADA	(−)	(−)	ITB^b^	(−)	ND	(−)	pleura
20	CD	F	35	36	11	IFX	(−)	(−)	ITB^b^	(−)	ND	(−)	Lung + endobronchus
21	CD	M	25	26	1	IFX	ND	(−)	No	(−)	ND	(+)	Lung

TNF, tumor necrosis factor; IBD, inflammatory bowel disease; UC, ulcerative colitis; CD, Crohn’s disease; LTBI, latent tuberculosis infection; TB, tuberculosis; TST, tuberculin skin test; IGRA, interferon gamma release assay; IFX, infliximab; ADA, adalimumab; ND, not done; ITB, intestinal tuberculosis; INH, isoniazid; RFP, rifampin; Ex, ex-smoker; LN, lymph node

^a^This patent was undergoing treatment for tuberculosis at the end of the study. All other patients were completely cured after tuberculosis treatment

^b^These patients with Crohn's disease had a history of medication for tuberculosis to identify intestinal tuberculosis

### Comparison between TB and non-TB groups

We used the Cox proportional hazard model to identify the risk of TB development in patients with IBD treated using anti-TNF therapy (Table 3). The mean age at which anti-TNF therapy was started was higher in the TB group (n = 21) than in the non-TB group (n = 1413; 39.57 vs. 31.22 years; *p* = 0.002). There were no significant differences between the two groups in sex, IBD type, smoking status, TB infection history and screening tests for LTBI. In patients who underwent LTBI screening, the positive rates of LTBI did not differ between the two groups (14.3% vs. 13.8%; *p* = 0.862). There were no significant differences in the incidence of TB between the LTBI-positive and LTBI-negative groups (*p* = 0.86).

**Table 3:** Risks of active tuberculosis infection during anti-TNF therapy, derived from a Cox proportional hazards model

	TB + group (n = 21)		TB- group (n = 1413)	Hazard ratio (95% CI)	*p* value
Age at anti-TNF (years)	39.57	31.22		1.041 (1.014–1.069)	0.002
Age of IBD diagnosis (years)	32.81	25.70		1.039 (1.012–1.067)	0.005
Female	9 (42.9)	485 (34.3)		1.286 (0.541–3.055)	0.570
IBD type				0.660 (0.254–1.714)	0.393
UC	6 (28.6)	323 (23.1)			
CD	15 (71.4)	1087 (76.9)			
History of smoking	10 (47.6)	433 (30.6)		2.094 (0.889–4.932)	0.091
Previous TB history	1 (4.7)	48 (3.4)		1.297 (0.174–9.665)	0.800
Screening for LTBI				1.170 (0.417–3.284)	0.765
IGRA + TST	15 (71.4)	951 (67.3)			
IGRA	5 (23.8)	361 (25.5)			
LTBI	3 (14.3)	195 (13.8)		0.896 (0.262–3.066)	0.862

TNF, tumor necrosis factor; IBD, inflammatory bowel disease; UC, ulcerative colitis; CD, Crohn’s disease; LTBI, latent tuberculosis infection; TB, tuberculosis

### Incidence rate of TB development according to age

**Fig. 2:**
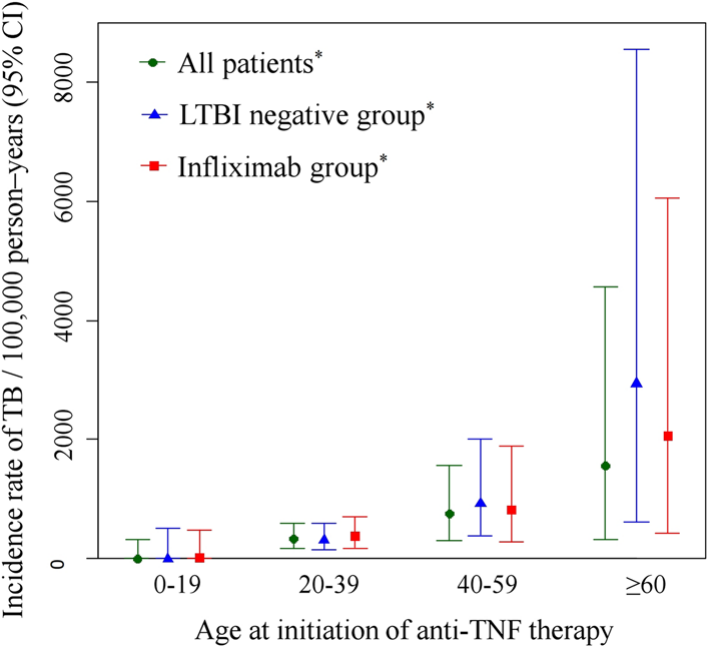
Incidence rates (per 100,000 person-years) of active tuberculosis infection according to age: all patients (circle), latent tuberculosis-negative group (triangle), infliximab group (square). *Linearity *p* < 0.05. TNF, tumor necrosis factor; TB, tuberculosis; LTBI, latent tuberculosis infection

The study subjects were divided into four age groups (0–19, 20–39, 40–59, and ≥ 60 years); the incidence rate of TB in each group was checked and cross-analysis was performed (Fig. 2). The incidence rate of TB in each age group was 0, 323.4, 756.6, and 1557.8 per 100,000 person-years, respectively, and the incidence rate increased linearly as the age increased (linearity *p* value < 0.001). The incidence of TB in LTBI-negative patients (n = 1135) was 0, 300, 915.6, and 2931.6 per 100,000 person-years, respectively (linearity *p* = 0.011), and the incidence of TB in patients receiving only infliximab (n = 939) was 0, 359.6, 798.7, and 2061.9 per 100,000 person-years, respectively (linearity *p* value < 0.001).

## Discussion

In Korea, the incidence of IBD is gradually increasing in all age groups, including elderly citizens [25]. Furthermore, the age of patients with IBD who are receiving treatment is increasing over time. Because of this trend, there is growing research interest in medication and its associated morbidities in older patients with IBD. As age increases, the production of T cells and the T cell-mediated response decreases, suppressing the formation of granulomas by mononuclear phagocytes activated by *Mycobacterium tuberculosis* infection and thus increasing the risk of active TB infection [26, 27]. The prevalence of nutritional deficiencies and other age-related diseases also increases, which can influence the morbidity of TB [27, 28]. In the present study, we identified a significant age difference between the TB group and the non-TB group of patients with IBD (39.57 vs. 31.22 years; *p* = 0.002), and there was a linear increase in the incidence rate of TB infection as the age at anti-TNF therapy initiation increased (Fig. 2).

It can be assumed that the age at anti-TNF therapy initiation is related to the incidence of TB in patients with IBD because of the high prevalence of LTBI-positivity in Korea in the older age group [23]. However, the incidence rate of TB development in LTBI-negative patients (n = 1135) is also higher in the older age group. It follows that age itself may be a risk factor for TB development in patients with IBD undergoing anti-TNF therapy, regardless of LTBI. In the infliximab-alone group, which contained the highest number of patients among the various anti-TNF therapy groups, the incidence rate increased linearly as the age group increased. However, in patients receiving other single or multiple anti-TNF agents, there was small sample size, so the prevalence of TB must be carefully interpreted with each anti-TNF agent. In addition, the present study was carried out in a country with an intermediate TB burden [23, 29]; therefore, caution should be exercised when applying the results to regions with different incidence rates.

In the present study, the incidence rate of TB development in patients with IBD treated with anti-TNF therapy was 366.73 per 100,000 person-years, and the prevalence was 1.46%. In another multicenter, retrospective study carried out in Korea, 16 cases of TB diagnosis were reported among 376 patients with IBD who underwent anti-TNF therapy (incidence rate 1997.4 per 100,000 person-years) [18]. Similarly, in a study that analyzed data from the National Health Insurance system in Korea, the incidence rate was 554.1 per 100,000 person-years [30]. As such, the incidence rate of TB development in the present study was lower than that in other studies. In the present study, most patients with IBD in whom anti-TNF therapy was planned were referred to TB specialists for LTBI screening and management, as well as continued follow-up observation. As a result, a high rate of LTBI evaluation (93%) was seen, and only eight out of the 198 patients did not complete TB prophylaxis, resulting in a high LTBI treatment completion rate (96%). In the aforementioned study, on the other hand, among the 30 LTBI-positive patients, 16 (53%) underwent TB prophylaxis [18]. Therefore, we believe that completion of LTBI treatment in LTBI-positive patients will lower the incidence rate and prevalence of TB development, and that a system for LTBI screening and management should be implemented to this end.

**Fig. 3:**
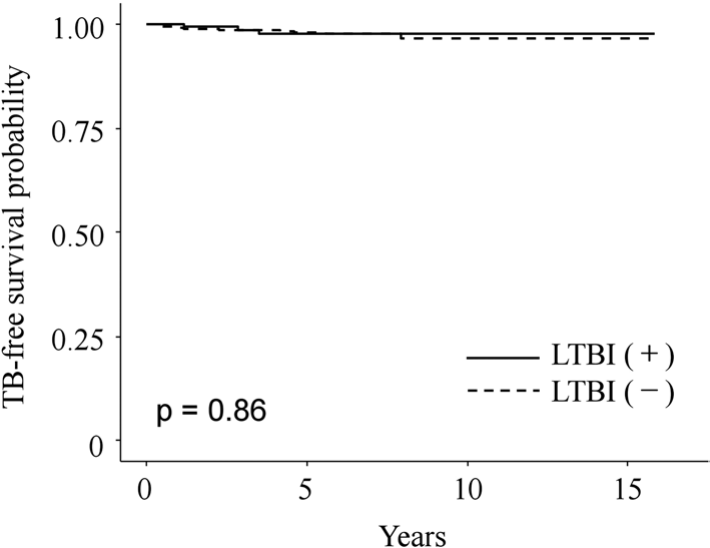
Kaplan–Meier curve for the incidence of active tuberculosis in patients with IBD who underwent anti-TNF therapy, based on presence of latent tuberculosis infection (LTBI), as follows: LTBI-positive (solid line), LTBI-negative (dotted line)

Another interesting finding was that the LTBI-positive rate did not show a significant difference between the TB and non-TB groups. Treatment of LTBI prior to immunosuppressant administration is thought to prevent active TB infection caused by LTBI reactivation [31, 32]. Unlike the results in the present study, a previous study conducted in Korea reported that LTBI could be a risk factor for the development of TB [18]. At the time the previous study was conducted, LTBI management was not systematically implemented and only a low percentage of patients completed TB prophylaxis. In the present study, the percentage of LTBI patients receiving TB prophylaxis increased to 96%. We believe that this difference may have significantly lowered the incidence of TB in LTBI-positive patients. As a result, the incidence of TB development in LTBI-positive patients was similar to that in LTBI-negative patients (Fig. 3). Moreover, a recent study reported the same 1-year incidence of TB in patients with IBD receiving anti-TNF therapy, regardless of LTBI status [33]. In another study, the expected reduction in the risk of TB development in the LTBI management group was 40–60% [34]. Thus, the results of the present study are in line with those of previous studies [33, 34] and confirm that LTBI is no longer a risk factor for TB development in patients undergoing anti-TNF therapy who have also undergone active LTBI management.

Although active work-up and management of LTBI was performed in the present study, the onset of TB was not completely suppressed. Anti-TNF therapy can increase the risk of LTBI reactivation and increase susceptibility to primary TB and exogenous reinfection [35, 36]. LTBI treatment can prevent TB development by LTBI reactivation, but it cannot prevent new active TB infection. Therefore, TB infection cannot be completely suppressed, even with active LTBI management; this is particularly important to consider in countries with a high TB burden. In addition, since the general indication for anti-TNF therapy in patients with IBD is non-response to universal treatments such as steroids and/or immunomodulators, many patients with IBD are also given these drugs when anti-TNF therapy is administered, which can suppress the immune response and increase the chances of TB infection. One study found that immunosuppression by steroids and/or immunomodulators when starting anti-TNF therapy can lead to a low white blood cell count, which may be a risk factor for TB in patients undergoing anti-TNF therapy [18]. Finally, the diagnostic tests for LTBI are not completely accurate. The TST and IGRA are more likely to produce false negative results if the patients are taking drugs such as steroids or immunomodulators, or if they have an immunodeficiency [37–39]. In addition, IGRA has a relatively low sensitivity compared with its high specificity; therefore, the possibility of false-negative results should be considered. For these reasons, we believe that several cases of active TB development were identified at the beginning of anti-TNF therapy despite a negative LTBI test. To confirm that the tests were reliable, we checked the HR of TB development in the TST plus IGRA group and the IGRA-only group; there was no significant difference between the groups in this regard (HR 1.170, *p* = 0.765). In immunosuppressed patients, researchers should be aware that the LTBI screening tests may be inaccurate and consider ways to increase the accuracy.

There were several limitations to the present study. First, LTBI evaluation was conducted in accordance with the Korean guidelines, so we did not consider several factors that can affect the test results. In particular, BCG vaccination can affect TST results and is mandatory in Korea [40], and immunosuppressants and various diseases can also affect result of TST and IGRA [37–39]. Second, although the age at which anti-TNF therapy was initiated correlated with the incidence rate of TB, the time taken for TB to develop varies; therefore, care must be taken when interpreting this result. Third, clinicians tended to prefer infliximab as the first anti-TNF agent in the present study. For this reason, we could not fully explore the effects of other anti-TNF agents or multiple drugs on the development of TB and the identification of risk factors because they were given to a smaller proportion of patients. Lastly, it was difficult to confirm the accurate history of the other immunosuppressants (except anti-TNF agents) that had been administered at other medical centers. Consequently, we could not take into consideration other immunosuppressants that can influence the morbidity of TB. Further research with a larger population is needed in the future.

## Conclusions

We confirmed that the incidence rate of TB increases linearly as the age at anti-TNF therapy initiation increases in patients with IBD. If anti-TNF therapy is administered to older patients, more rigorous monitoring for active TB development may be required. In addition, active LTBI screening and management may lower the incidence of TB development. Therefore, plans for monitoring TB development must be established, considering these characteristics.

## Data Availability

The data underlying this article cannot be shared publicly due to the privacy of individuals that participated in the study, but are available from the corresponding author (Sang Hyoung Park) on reasonable request.

## Abbreviations

CD: Crohn's disease
IBD: Inflammatory bowel disease
IGRA: Interferon gamma release assay
LTBI: Latent tuberculosis infection
PPD: Purified protein derivative
TST: Tuberculin skin test
TB: Tuberculosis
TNF: Tumor necrosis factor
UC: Ulcerative colitis
